# Photoactivation in Myxobacterial Phytochrome: Early Events

**DOI:** 10.1063/4.0000959

**Published:** 2025-10-27

**Authors:** Emina A Stojkovic, Tek N Malla, Sebastian Westenhoff, Marius Schmidt

**Affiliations:** 1 Northeastern Illinois University, Chicago, IL, USA; 2 University of Wisconsin-Milwaukee, Milwaukee, WI, USA; 3 Uppsala University, Uppsala, Sweden, Sweden

## Abstract

Myxobacteria are non-photosynthetic, soil bacteria distinguished by a multicellular stage in their life cycle known as fruiting bodies. Stimulated by light, fruiting bodies from myxobacteria may rely on phytochrome function as protein photoreceptor responsible for the perception of red-light. The molecular mechanism how light is perceived and converted to a physiological response is unknown. Here, time-resolved serial femtosecond crystallographic (TR-SFX) experiments were conducted on microcrystals of the *Stigmatella aurantiaca* bacteriophytochrome 2 (SaBphP2). Initial events of the Z to E isomerization reaction of biliverdin, the covalently bound, open-chain tetrapyrrole, were explored. At 3 ps after light activation, the BV ring-D assumes a configuration needed for the isomerization. At 100 ps, a mixture of BV in the Z or E configuration is observed in subunit A, while in the other subunit the BV chromophore remains in the Z configuration. In conjunction with prior results, these BV structures shine light on the molecular mechanism of phytochrome activation involved in the photomorphogenesis of the myxobacteria and lay the molecular foundation to explain physiological responses to red light in other bacteria.

